# Reciprocal Regulation of Axonal Filopodia and Outgrowth during Neuromuscular Junction Development

**DOI:** 10.1371/journal.pone.0044759

**Published:** 2012-09-05

**Authors:** Pan P. Li, Jie J. Zhou, Min Meng, Raghavan Madhavan, H. Benjamin Peng

**Affiliations:** Division of Life Science, State Key Laboratory of Molecular Neuroscience, The Hong Kong University of Science and Technology, Clear Water Bay, Hong Kong, China; Virginia Commonwealth University Medical Center, United States of America

## Abstract

**Background:**

The assembly of the vertebrate neuromuscular junction (NMJ) is initiated when nerve and muscle first contact each other by filopodial processes which are thought to enable close interactions between the synaptic partners and facilitate synaptogenesis. We recently reported that embryonic Xenopus spinal neurons preferentially extended filopodia towards cocultured muscle cells and that basic fibroblast growth factor (bFGF) produced by muscle activated neuronal FGF receptor 1 (FGFR1) to induce filopodia and favor synaptogenesis. Intriguingly, in an earlier study we found that neurotrophins (NTs), a different set of target-derived factors that act through Trk receptor tyrosine kinases, promoted neuronal growth but hindered presynaptic differentiation and NMJ formation. Thus, here we investigated how bFGF- and NT-signals in neurons jointly elicit presynaptic changes during the earliest stages of NMJ development.

**Methodology/Principal Findings:**

Whereas forced expression of wild-type TrkB in neurons reduced filopodial extension and triggered axonal outgrowth, expression of a mutant TrkB lacking the intracellular kinase domain enhanced filopodial growth and slowed axonal advance. Neurons overexpressing wild-type FGFR1 also displayed more filopodia than control neurons, in accord with our previous findings, and, notably, this elevation in filopodial density was suppressed when neurons were chronically treated from the beginning of the culture period with BDNF, the NT that specifically activates TrkB. Conversely, inhibition by BDNF of NMJ formation in nerve-muscle cocultures was partly reversed by the overexpression of bFGF in muscle.

**Conclusions:**

Our results suggest that the balance between neuronal FGFR1- and TrkB-dependent filopodial assembly and axonal outgrowth regulates the establishment of incipient NMJs.

## Introduction

The development of the vertebrate neuromuscular junction (NMJ) is widely studied for understanding the cellular and molecular control of synaptogenesis [1]. During NMJ assembly the tip of a motor neuron – the axonal growth cone – approaches and contacts muscle and then transforms into a presynaptic terminal specialized for secreting the neurotransmitter acetylcholine (ACh). The hallmark of this differentiation process is the accumulation of synaptic vesicles and mitochondria in the nerve terminal [1], [2]. Concomitantly, morphological and molecular changes in the muscle fiber at sites contacted by nerve generate a postsynaptic domain that detects nerve-released ACh unfailingly [1]. The most critical step in postsynaptic differentiation is the clustering of acetylcholine receptors (AChRs) in the muscle membrane apposed to presynaptic terminals, which produces an AChR density of ∼10,000/µm^2^ at adult NMJs, one thousand-times higher than in the extrasynaptic membrane [3].

Synaptogenic changes at the developing NMJ are induced and regulated by nerve- and muscle-secreted molecules, many of which are associated with a synaptic basal lamina that appears between the nerve and muscle membranes as the NMJ matures. The basal lamina anchors growth factors, enzymes and other synapse-associated molecules with distinct pre- and post-synaptic functions, such as agrin, neuregulins, laminins, Wnts, acetylcholinesterase, and several members of the fibroblast growth factor (FGF) family [4]–[9]. Heparan sulfate proteoglycans (HSPGs) are important components of the synaptic basal lamina [10], [11] and they serve as storage sites for growth factors [12], [13]. Our group has previously studied the functions of many HSPG-associated molecules produced by nerve and muscle, including basic FGF (bFGF), heparin-binding growth associated molecule and the closely related molecule midkine, and hepatocyte growth factor [6], [14]–[17]. The purpose of the current study was to further investigate the molecular regulation of presynaptic differentiation during the earliest stages of NMJ formation.

Interactions between growing motor axons and their muscle targets are first mediated by motile filopodia, the thin and dynamic actin-rich processes that help cells sense environmental signals [18]–[20]. Filopodia are abundant in neuronal growth cones and aid axonal pathfinding [18], [21], [22], although filopodia also form along axonal shafts [23], [24]. In CNS neurons filopodial growth along dendrites is robust during synaptogenesis [25], and at the developing NMJ filopodia are extended by both nerve and muscle [14], [26]–[28]. We found that blocking filopodial assembly in either muscle or nerve hindered NMJ development in Xenopus nerve-muscle cocultures [14], [26], and moreover that bFGF produced by muscle activated FGFR1 in neurons to induce filopodia which facilitate NMJ establishment [14]. Intriguingly, earlier work from our group showed that neuronal growth and survival are enhanced but NMJ formation is inhibited in response to neurotrophins (NTs) [29] which are also made by neuronal targets [30]–[32]. One of these NTs, brain-derived neurotrophic factor (BDNF), produces its effects on motor neurons by activating the tropomyosin-related kinase B (TrkB) receptor [33], [34]. Axonal outgrowth and filopodial formation are thus opposing events that are regulated by different signals.

Because NTs/BDNF and bFGF/FGFR1 differently influence axonal behavior and NMJ establishment [14], [29], here we investigated how they together effect presynaptic changes. We manipulated bFGF/FGFR1 and BDNF/TrkB in Xenopus spinal neurons and muscle cells and quantified axonal growth, filopodial generation, the extension of neuronal filopodia towards muscle, and NMJ assembly. Our results suggest that FGFR1-dependent differentiation of neurons promotes NMJ formation by counterbalancing TrkB-stimulated axonal outgrowth.

## Results

### Expression of FGFR1 and TrkB in Xenopus nerve and muscle

**Figure 1:**
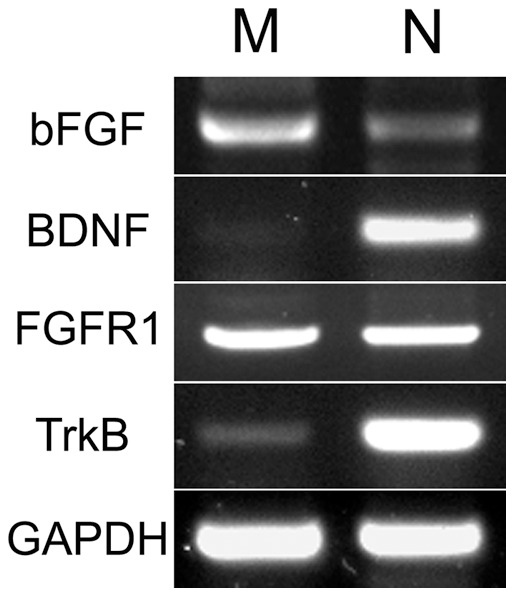
Expression of bFGF/FGFR1 and BDNF/TrkB in Xenopus nerve and muscle. These agarose gel photographs show RT-PCR-generated fragments from mRNAs of bFGF, BDNF, FGFR1, TrkB and GAPDH in neural tubes (N) and myotomes (M) isolated from Xenopus embryos. GAPDH was used as an amplification and loading control.

Previously we studied the effects of NTs/BDNF and bFGF signaling separately on NMJ formation using Xenopus nerve-muscle cocultures [14], [29]. To begin to address how these factors may control presynaptic differentiation jointly, we first examined the relative expression of BDNF/TrkB and bFGF/FGFR1 in nerve and muscle. Several commercially available antibodies against these proteins failed to label specifically their counterparts in Xenopus, so we sought to examine their mRNA levels. RT-PCR assays using neural tubes and myotomal muscle tissue isolated from Xenopus embryos showed that bFGF mRNA was present at a much higher level in myotomes than in neural tubes (Figure 1), consistent with our earlier immunoblotting and immunolabeling results [14]. The mRNA for bFGF's receptor, FGFR1, was expressed in nearly equal amounts in myotomes and neural tubes, but mRNAs for BDNF and TrkB were mainly found in neural tissue. Here GAPDH mRNA served as a control for PCR amplification and gel loading (Figure 1).

### Activation of endogenous TrkB in spinal neurons

**Figure 2:**
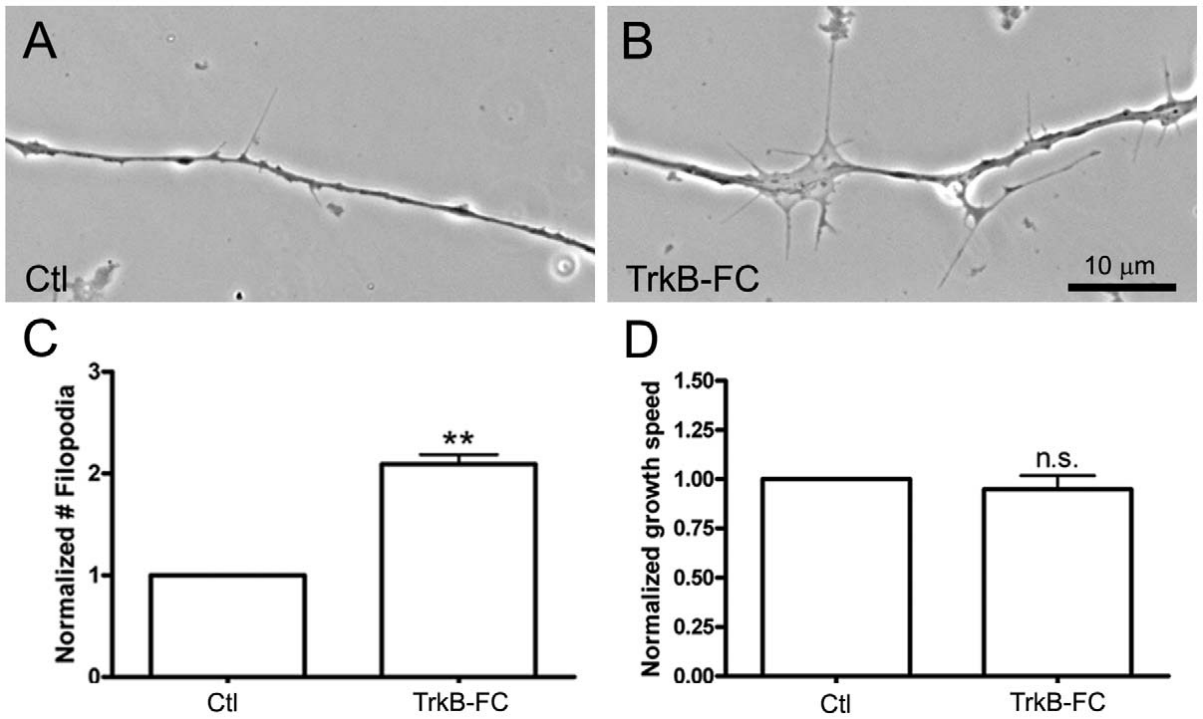
Influence of BDNF-depletion on the formation of axonal filopodia. Spinal neurons were incubated in (A) control medium or (B) medium containing 1 µg/ml TrkB-FC. Axonal filopodial formation was examined 18 h after plating the neurons. Bath application of TrkB-FC enhanced the formation of axonal filopodia, which is quantified in terms of filopodial densities (C). The density of filopodia (#filopodia/10 µm axon) in TrkB-FC-treated neurons was normalized against that in control neurons. In the presence of TrkB-FC, no significant difference in the speed of axonal outgrowth was observed (D). Mean and SEM shown; t test: **p<0.01, compared with untreated Ctl.

As NTs are generally considered to be target-derived molecules, the expression of BDNF in neural but not muscle tissue was puzzling. We thus investigated if TrkB in spinal neurons can be activated through autocrine BDNF signaling or can become auto-activated in the absence of target muscle. Labeling with an anti-phospho-TrkB antibody did not give unambiguous evidence of TrkB autophosphorylation (thus activation) in cultured spinal neurons, presumably due to the lack of antibody specificity. We therefore tested for functional TrkB activation by pharmacological approaches. First, to examine if there is autocrine activation of TrkB – as a result of the secretion of BDNF from spinal neurons themselves – TrkB-Fc was used as a scavenger to remove BDNF from the medium. Previously we found that cessation of axonal growth upon target recognition is accompanied by increased generation of axonal filopodia [14]. Thus, we examined the effect of TrkB-Fc on the number of axonal filopodia in this study. As shown in Figure 2 (A–C), nearly twice as many filopodia were observed in TrkB-Fc-treated neuronal cultures than in untreated cultures. In addition, the speed of axonal advance was assessed from time-lapse recordings: as shown in Figure 2D, addition of TrkB-Fc to cultures produced no significant change in axonal outgrowth speed compared to the addition of control medium.

**Figure 3:**
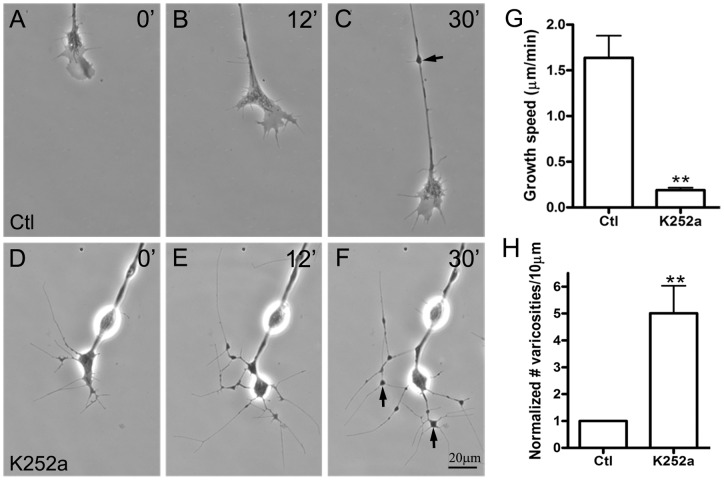
Effect of Trk inhibition on axonal growth. Spinal neurons were incubated in control medium (A–C) or medium containing 500 nM K252a (D–F). The growth speeds of axons were measured from images taken during time-lapse recordings. Treatment with K252a led to a significant reduction in axonal growth speed (G) and also to the elaboration of the distal end of the axon into a web of interconnecting neurites and varicosities within 30 min. Although varicosities were also observed along control axons and were often associated with filopodia (arrow in C), they were much more numerous within the distal neuritic web generated after K252a treatment (arrows in F and quantified in H). Mean and SEM shown; t test: **p<0.01, compared with untreated Ctl.

Second, axonal growth was examined in the presence of the pan-Trk inhibitor K252a [35]. As shown in Figure 3, addition of this drug to pure nerve cultures caused a dramatic retardation in axonal growth and triggered significant changes in the morphology of the growth cone (compare control axon in A–C to the treated one in D–F). Together with the cessation of growth (Figure 2G), the growth cone and the distal part of the axon were observed to develop into a neuritic web that encompassed varicosities at intersections of neurites (Figure 2F arrows), which were enriched in synaptic vesicles and mitochondria – as shown by labeling with an antibody against synapsin I (Figure S1, C–D) and live-cell labeling with the mitochondrial probe MitoTracker (Figure S1, K–L). Live imaging of neurons expressing GFP-conjugated synaptophysin also confirmed the clustering of synaptic vesicles within these varicosities in K252a-treated cultures (Figure S1, G–H). Although filopodia, which were often associated with varicosities along the axon (Figure 3C, arrow, also see [14]), were observed on axons in control neuron cultures, they were mainly manifested as interconnecting neurites in K252a-treated neurons (Figure 3D). We thus compared the number of varicosities as a measure of filopodial emanation (in control neurons) versus neuritic arborization (in K252a-treated neurons) and found that, relative to control, K252a-treatment triggered a large increase in varicosities (Figure 3H). Such a change was also clearly evident upon labeling neuronal cultures with markers for synaptic vesicles (Figure S1). This effect was specific for the inhibition of Trk with K252a as inhibitors against other signaling molecules, including H89 for PKA, KN-93 for CaMKII, Gö6983 for PKC and SU5402 for FGF-receptor, did not reproduce these morphological changes in growing spinal axons (data not shown). These results suggested that endogenous Trk receptor is activated during the growth stage of spinal neurons.

These two lines of data together offer functional evidence that TrkB in spinal neurons can become activated through autocrine and/or auto-activation mechanisms. As the effect of K252a on axonal outgrowth and filopodia/neuritic arbor was much stronger than that of TrkB-Fc, the auto-activation seems to be the major mechanism. Because K252a, like most kinase inhibitors, can also affect signaling pathways other than that initiated by Trk, experiments were also conducted to specifically manipulate TrkB signaling with molecular methods. As described below, results from those studies confirmed these pharmacological data.

### Differential effects of bFGF/FGFR1- and BDNF/TrkB-signaling on filopodial assembly and neuronal growth

**Figure 4:**
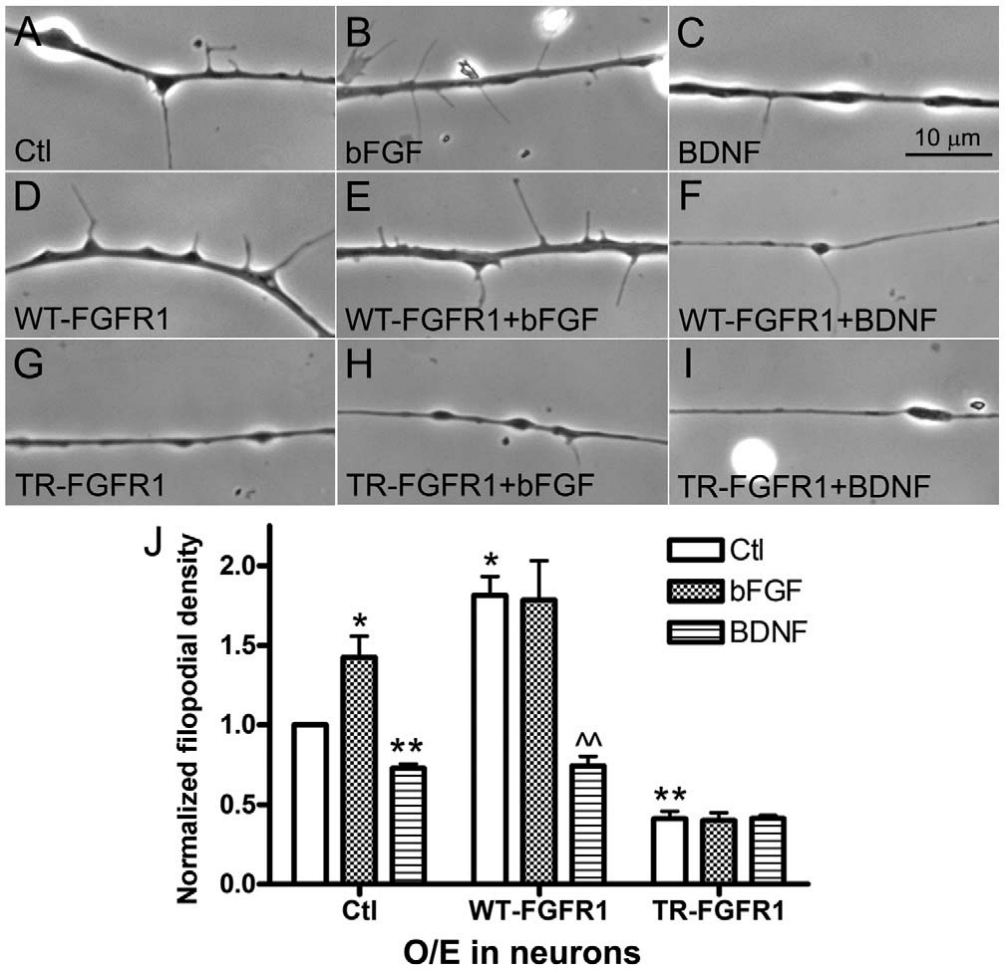
Opposite effects of bFGF and BDNF signaling on filopodial formation in spinal neurons. Spinal neurons were incubated in control medium (A) or medium containing (B) bFGF or (C) BDNF. Filopodial assembly was enhanced and suppressed by bFGF and BDNF respectively (J). Neurons expressing WT-FGFR1 or TR-FGFR1 were also maintained in control medium (D, G) or medium containing bFGF (E, H) or BDNF (F, I). WT-FGFR1 expression elevated the basal filopodial density in neurons, and this was not increased by bFGF-addition but was suppressed by BDNF-treatment (J). TR-FGFR1 expression blocked both basal and bFGF-induced formation of filopodia in neurons, and BDNF did not further reduce filopodial growth (J). The density of filopodia in control neurons (A) was used for normalizing all other filopodial density values. Mean and SEM shown; t test: *p<0.05 and **p<0.01, compared with untreated Ctl; ??p<0.01, compared to untreated WT-FGFR1-neurons.

In Xenopus nerve-muscle cocultures, bFGF promotes presynaptic differentiation [14], [36] and NTs support neuronal survival and growth [29]. Using spinal neurons cultured in the absence of muscle cells, here we compared how bFGF and BDNF affect filopodial induction in the axons, a differentiation step which fosters synaptogenesis [14]. Overnight exposure of wild-type neurons to bFGF (100 ng/ml) enhanced filopodial formation, whereas exposure to BDNF (10 ng/ml) suppressed filopodial assembly (Figure 4, A–C). Relative to control neurons, bFGF- and BDNF-treated neurons had ∼50 % higher and ∼30 % lower filopodial densities respectively (Figure 4J).

**Figure 5:**
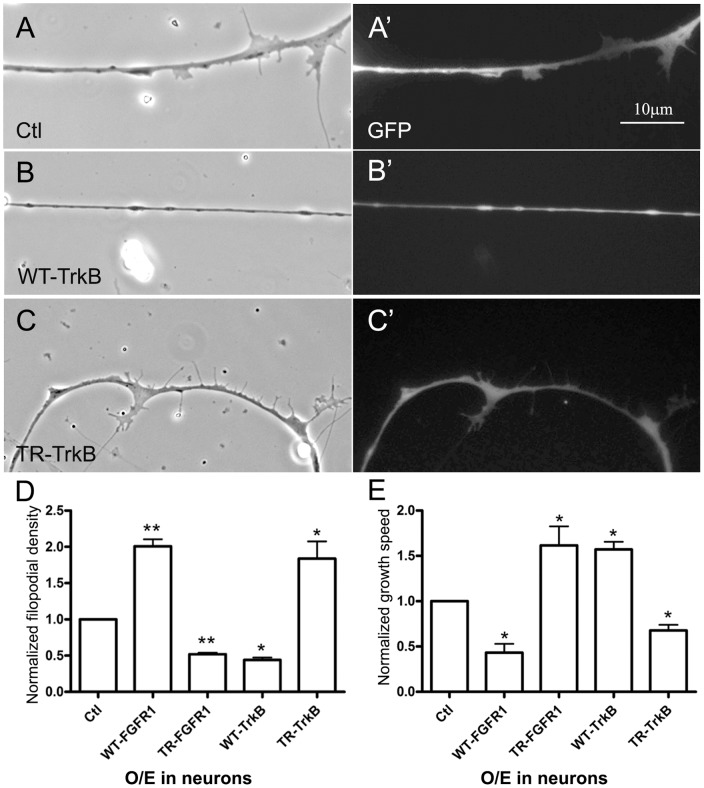
Differential control of filopodial assembly and axonal growth by neuronal FGFR1 and TrkB signaling. Compared to control (GFP) neurons (A and A'), WT-TrkB-neurons grew fewer filopodia (B and B') and TR-TrkB-neurons grew more filopodia (C and C'); panels A–C and A'–C' show corresponding phase-contrast and GFP images. In panel D the filopodial densities calculated for these neurons are normalized relative to the density in GFP-neurons and are compared to those obtained for neurons expressing active and inactive FGFR1 proteins (Figure 4). E. Axonal growth speeds of neurons expressing FGFR1 or TrkB proteins were measured by time-lapse imaging and normalized relative to the growth of GFP-neurons. Overexpression of WT-FGFR1 or TR-TrkB slowed axonal growth, whereas expression of TR-FGFR1 or WT-TrkB sped it up. Mean and SEM shown; t test: *p<0.05, compared with Ctl.

Next, bFGF and BDNF were added to spinal neurons expressing wild-type FGFR1 (WT-FGFR1) (Figure 4, D–F) or truncated FGFR1 (TR-FGFR1) (Figure 4, G–I). Here and in other experiments described below, exogenous proteins were expressed in neurons (or muscle cells) by injecting Xenopus embryos with their mRNAs (mixed with GFP mRNA) and then culturing neurons (or muscle cells) from the injected embryos. GFP fluorescence was used to identify cells that expressed these exogenous proteins (as shown in the examples in Figure 5), and the functionality of the constructs was assessed in HEK293T cells: overexpression of WT-FGFR1 in HEK293T cells led to the autophosphorylation of this receptor in the absence of bFGF, but the expression of kinase-deleted TR-FGFR1 did not (Figure S2A).

Introduction of WT-FGFR1 into neurons almost doubled the basal filopodial density (Figure 4D) as before [14], and adding bFGF did not enhance this further (Figure 4E). Coupled with the results from heterologous cells (above) this suggested that overexpressed WT-FGFR1 was mostly activated and hence unresponsive to exogenous bFGF. Interestingly, treatment with BDNF more than halved the density of filopodia in WT-FGFR1-neurons (Figure 4F), suggesting that the stimulation of TrkB in neurons by BDNF can overcome the filopodium-inducing signal generated by WT-FGFR1. In contrast to WT-FGFR1, expression of TR-FGFR1 blocked both basal (Figure 4G) and bFGF-dependent filopodial growth (Figure 4H) and BDNF-treatment did not affect the already low filopodial density (Figure 4I). These results are quantified in Figure 4J.

BDNF activates signaling by the TrkB receptor [34]. We tested TrkB's role in the inhibition of filopodial formation by expressing wild-type TrkB (WT-TrkB) or truncated TrkB (TR-TrkB) in Xenopus spinal neurons. The TR-TrkB protein lacks the intracellular kinase domain and suppresses neuronal responses to BDNF [37]. These constructs were again tested in heterologous cells: we observed auto-activation of WT-TrkB (as shown by its tyrosine phosphorylation) when it was expressed in HEK293T cells, but this was not evident when the cells were transfected with the cDNA for TR-TrkB (Figure S2B). Supporting our BDNF results, relative to control GFP-neurons (Figure 5, A and A') WT-TrkB-expressing neurons developed fewer filopodia (Figure 5, B and B') and TR-TrkB-expressing neurons grew more filopodia (Figure 5, C and C'). Comparison of these filopodial densities (in Figure 5D) with those from FGFR1 experiments above shows that the expression of WT-TrkB, like that of TR-FGFR1, reduced filopodial assembly, and that the introduction into neurons of “dominant-negative” TrkB enhanced filopodial growth, like WT-FGFR1 overexpression.

The decrease in filopodial density produced by TR-FGFR1 or WT-TrkB expression could have resulted from either poor initiation of filopodial growth or a reduction in the lifetime of these dynamic structures. Thus neurons expressing these exogenous molecules were examined by time-lapse imaging. We found that the rate of filopodial assembly was suppressed under the influence of these two receptor constructs, much as in our recent study showing that a mutant form of p120 catenin that fails to inhibit RhoA blocks the initiation of new filopodia [38].

Because axonal filopodial formation and outgrowth are both processes that depend on membrane addition [39], [40], we tested whether FGFR1 and TrkB also affect the rate of axonal advance differently. Compared to control neurons, neurons expressing WT-FGFR1 or TR-TrkB grew 40–60 % slower while those expressing TR-FGFR1 or WT-TrkB grew ∼50 % faster (Figure 5E), suggesting that inhibition of FGFR1 or activation of TrkB promoted neuronal growth and activation of FGFR1 or inhibition of TrkB impeded growth. Thus, FGFR1 and TrkB oppositely regulated filopodial assembly as well as axonal growth in neurons.

### Influence of TrkB and FGFR1 on filopodial extension by neurons towards muscle

**Figure 6:**
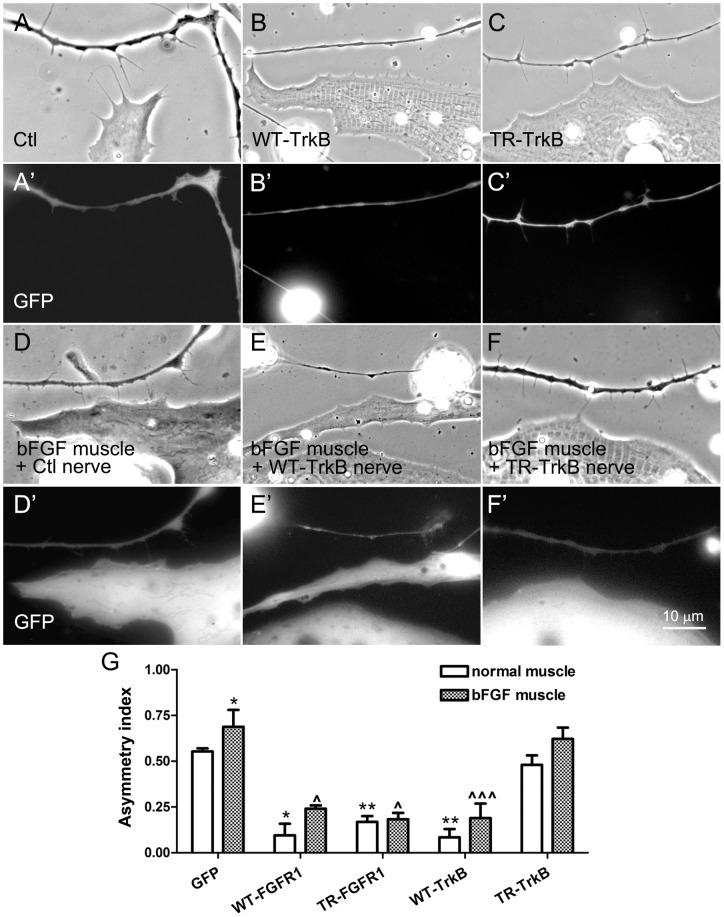
The influence of TrkB signaling on filopodial extension by neurons towards muscle. Nerve-muscle cocultures were prepared using neurons expressing GFP (A and A'), WT-TrkB (B and B') or TR-TrkB (C and C'). WT-TrkB-neurons, unlike control neurons, displayed little bias in the extension of filopodia towards muscle, but the TR-TrkB-neurons, which grew more filopodia than GFP-neurons, were able to send out filopodia preferentially in the direction of muscle. (D–F') On muscle cells expressing GFP plus bFGF, neurons expressing GFP (D and D') or TrkB proteins (E–F') were seeded. GFP-neurons extended even more filopodia towards muscle cells overexpressing bFGF (D and D') than towards normal muscle cells (A and A'). WT-TrkB-neurons once again grew fewer filopodia than GFP-neurons, but the bFGF-overexpressing muscle cells induced more filopodia in WT-TrkB-neurons (E and E') than control muscle cells (above). TR-TrkB-neurons also extended more filopodia towards bFGF-expressing muscle cells (F and F') than towards control cells. (G) Calculation of AI values for these cocultures as well as for those cultures in which neurons expressed FGFR1 proteins (pictures not shown). Asymmetric distribution of filopodia was slightly improved in neurons expressing WT-TrkB, TR-TrkB and WT-FGFR1, but not TR-FGFR1. Mean and SEM shown; t test: *p<0.05 and **p<0.01, compared to cocultures between Ctl neurons and normal muscle cells; ?p<0.05 and ???p<0.001, compared to cocultures made between Ctl neurons and bFGF-overexpressing muscle cells.

Xenopus spinal neurons approaching muscle cells extend more filopodia from their muscle-facing side than from the opposite side, indicating that these filopodia are induced by factors presented by muscle, such as bFGF [14]. To investigate how TrkB regulates muscle-dependent filopodial extension by neurons, spinal neurons expressing GFP (Figure 6, A and A'), WT-TrkB (Figure 6, B and B') or TR-TrkB (Figure 6, C and C') were cocultured with 7d-old muscle cells. The growth of neuronal filopodia towards muscle cells was quantified as asymmetry index values (AIs; Figure 6G) using nerve-muscle pairs as previously described [14]. Neurons expressing WT-TrkB extended filopodia less preferentially towards muscle than GFP-neurons, suggesting that the activation of TrkB not only reduced the spontaneous formation of filopodia but also inhibited the induction of neuronal filopodia by muscle. In contrast, neurons expressing TR-TrkB retained the ability to grow more filopodia towards muscle cells than away from them (Figure 6G). Next we examined the behavior of neurons expressing exogenous TrkB proteins when they approached muscle cells overexpressing bFGF. In this experiment, muscle cells isolated from bFGF-overexpressing embryos were plated on glass coverslips and 7 days later FGFR1 or TrkB-expressing neurons were seeded onto the muscle cells to prepare nerve-muscle cocultures. We observed that expression of excess bFGF in muscle increased the asymmetric growth of filopodia in control neurons (Figure 6, D and D'), as before [14], and also improved filopodial extension towards muscle by WT-TrkB neurons (Figure 6, E and E') and TR-TrkB-neurons (Figure 6, F and F'). On the other hand, bath application of BDNF in wild-type nerve-muscle cocultures reduced, presumably through neuronal TrkB activation, the preferential growth of axonal filopodia towards muscle cells (Figure S3).

In parallel experiments we cocultured WT-FGFR1- and TR-FGFR1-neurons with control and bFGF-overexpressing muscle cells (pictures not shown; AIs presented in Figure 6G). WT-FGFR1- and TR-FGFR1-neurons had more and less filopodia than GFP-neurons, respectively, and both displayed a marked decrease in the extension of filopodia preferentially towards muscle cells, much as in our previous work [14]. Overexpression of bFGF in muscle slightly increased the growth of filopodia towards muscle in WT-FGFR1- but not in TR-FGFR1-neurons (Figure 6G), which again indicated that normal FGFR1 signaling in neurons is needed for bFGF to elicit filopodial growth.

These results suggest that TrkB-dependent neuronal growth opposes FGFR1-mediated filopodial induction, but that bFGF (and perhaps other factors) from muscle can partly counter this to elicit filopodial growth in approaching neurons.

### Positive and negative regulation of NMJ assembly by bFGF and BDNF

**Figure 7:**
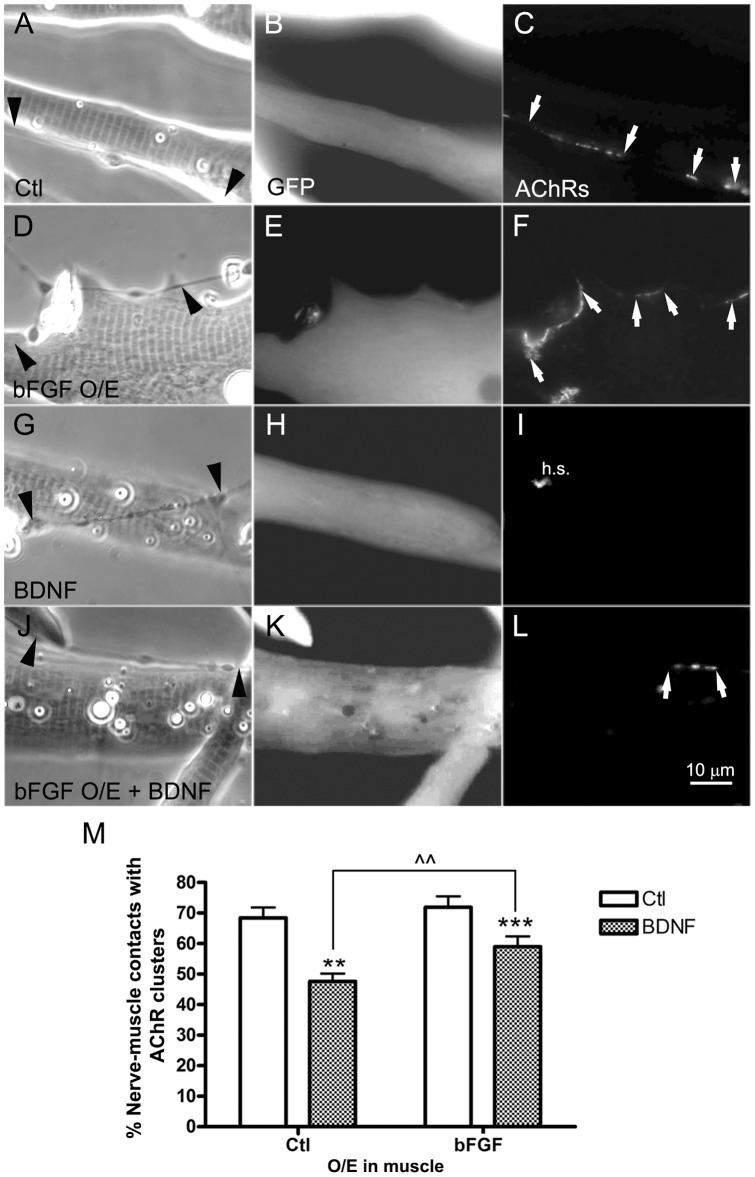
Reciprocal regulation of NMJ formation by bFGF/NT-signaling. Normal spinal neurons were cocultured with muscle cells expressing GFP (A–C and G–I) or GFP plus bFGF (bFGF O/E; D–F and J–L) and maintained in control medium (A–F) or medium with added BDNF (G–L). Neurons induced AChR clusters equally well in GFP- and bFGF-muscle cells (C, F), and BDNF-treatment inhibited synaptic AChR clustering in both cases (I, L). The inhibitory effect of BDNF, however, was weaker when muscle cells expressed excess bFGF, and in these cases nerve-muscle contacts with AChR were more readily found (L). Muscle cells in which nerves induced new AChR clusters lacked spontaneously occurring AChR aggregates (also called hot-spots); when AChR aggregation at innervation sites was compromised, AChR hot-spots were retained (h.s. in panel I). Synaptogenesis was quantified in terms of the percentages of nerve-muscle contacts with AChR clusters (M). Mean and SEM shown; t test: **p<0.01 and ***p<0.001, compared to cocultures with normal muscle cells; ??p<0.01, compared with BDNF-treated cocultures using bFGF-overexpressing muscle cells. Arrowheads point to nerve tracks, arrows to nerve-induced AChR clusters.

Inhibition by NTs/BDNF of NMJ formation [29] and filopodial induction in neurons (this study) are contrasted by the positive effect of muscle-bFGF on filopodial growth in neurons and on synaptogenesis [14]. To test if bFGF provided by muscle can overcome the suppressive effect of NTs on NMJ assembly, wild-type neurons were cocultured with muscle cells expressing GFP (Figure 7, A–C and G–I) or GFP plus bFGF (Figure 7, D–F and J–L). These cultures were maintained overnight in medium without or with added BDNF (10 ng/ml) and then labeled with R-BTX to mark muscle AChR clusters and quantify synaptogenesis. In cultures using GFP-muscle cells, AChRs were clustered at ∼70 % of nerve-muscle contacts (Figure 7, C and M), but the addition of BDNF inhibited this nerve-induced AChR aggregation by ∼35 % (Figure 7, I and M). When muscle cells overexpressing bFGF were used in cocultures, in the absence of BDNF again ∼70 % of nerve-muscle contacts had AChR clusters (Figure 7, F and M). Importantly, treatment with BDNF now reduced nerve-induced AChR aggregation by <20 % (Figure 7, L and M). Thus, overexpression of bFGF in muscle, which induces neuronal filopodia, partially rescued NMJ development in the presence of added BDNF, which triggers neuronal growth and suppresses filopodial assembly.

## Discussion

This study investigated the influence on presynaptic differentiation of two factors that separately bolster and block NMJ assembly. Our results suggest that bFGF and BDNF activate their receptors FGFR1 and TrkB in spinal neurons to enhance filopodial extension and neuronal growth respectively. Overexpression of TrkB in neurons produced effects akin to the inactivation of FGFR1, promoting axonal growth but suppressing filopodial formation and reducing filopodial extension towards muscle. The latter inhibitory effect was partly alleviated by the forced expression of bFGF in muscle, which stimulates FGFR1 signaling in neurons to elicit filopodial growth [14]. Importantly, overexpression of bFGF in muscle improved the formation of NMJs in nerve-muscle cocultures treated with BDNF. BDNF increased neuronal growth but blocked synaptogenesis like a NT-cocktail used before [29]. We therefore propose that activation of neuronal FGFR1 by muscle-derived bFGF during early nerve-muscle interaction helps offset BDNF/TrkB signaling to enable growing neurons to establish synaptogenic contacts with muscle.

NTs support the survival of many types of neurons, including motor neurons. The NT family includes nerve growth factor (NGF), BDNF, NT-3 and NT-4/5 that act through Trk receptors and p75 NT-receptor (p75NTR). The “low-affinity” p75NTR aids the selective binding of different NTs to specific Trks, although p75NTR signaling can also lead to neuronal death; in contrast, stimulation of the Trk tyrosine kinases promotes neuronal growth and survival [41]–[45]. NGF activates TrkA in sensory and sympathetic neurons [46], and BDNF and NT-4/5 activate TrkB in motor neurons [33], [34]. TrkC is the receptor for NT-3, which is present at low levels at the NMJ [47]. This study and our previous work [29] have together shown that BDNF promotes the survival and growth of Xenopus spinal neurons, that TrkB mRNA is expressed by Xenopus neural tubes, and that the expression of WT-TrkB in spinal neurons mimics the stimulation of neurons by NTs/BDNF. Although continued perturbation of NT signaling can compromise neuronal survival, in our experiments the TR-TrkB-expressing Xenopus spinal neurons did not show signs of apoptosis, presumably because they were studied at an early stage (within 18 h after plating). Similarly, neurons expressing WT-FGFR1, TR-FGFR1 or WT-TrkB were also healthy during the period of our experiments.

Unlike the addition of BDNF or expression of WT-TrkB, introduction of TR-TrkB into neurons reduced neuronal growth and generated filopodia. Neurons treated with bFGF or overexpressing FGFR1 likewise grew slowly and extended more filopodia than control neurons. In accord, filopodia are induced along the dendrites of hippocampal neurons by the expression of truncated TrkB receptors TrkB.T1 and TrkB.T2 [48], and bFGF stimulates filopodial formation in cortical neurons [49]. However TrkB signaling may not regulate outgrowth in all neuronal types: in retinal ganglion cells (RGCs), BDNF promoted axonal branching [50], but RGCs expressing the dominant-negative TrkB receptor extended normally to the tectum, indicating unperturbed outgrowth, although they failed to branch properly once in the target area [51].

Here, in Xenopus spinal neurons, TrkB signaling sustained a “growth-state” marked by faster axonal elongation and reduced filopodial assembly coupled with diminished muscle-directed filopodial extension and synaptogenesis. Bath-applied BDNF also lowered the density of filopodia in neurons overexpressing WT-FGFR1. Conversely, bFGF/FGFR1 signaling helped neurons enter a “synaptogenic-state” in which axonal advance was slower and filopodial growth more robust. Notably, overexpression of bFGF in muscle was able to dampen BDNF's inhibitory effect on NMJ development. Thus, the relative levels of TrkB and FGFR1 signaling in a neuron arriving at its targets may either maintain the neuron in a growth-state or switch it to a synaptogenic state, with higher TrkB activity favoring the former and stronger FGFR1 stimulation the latter.

How TrkB/FGFR1 signaling might be balanced is unknown but two possibilities are suggested by previous studies. First, the adapter proteins Shc and FRS2 bind to the same tyrosine-phosphorylation site in TrkA and may compete in doing so [52]. Phosphorylation of Shc bound to TrkA and the subsequent recruitment by Shc of Grb2/Sos results in Ras-dependent MAP kinase activation that is transient and promotes growth; in contrast, phosphorylation of FRS2 leads to prolonged activation of MAP kinase and serves as a differentiation signal [52]. TrkB, like TrkA, binds to Shc [41], so the recruitment and activation of Shc by TrkB could maintain spinal neurons in a growth-state. FRS2 is a major substrate of FGFRs and can constitutively bind FGFR1 [53], which may potentially activate FRS2 to boost neuronal differentiation.

Second, tyrosine phosphorylation of FRS2 downstream from FGFR1 might activate the tyrosine phosphatase Shp2 and reduce TrkB-signaling. Shp2 has been reported to negatively regulate BDNF-dependent phosphorylation of proteins in CNS neurons [54]. Shp2 is also activated upon binding to another tyrosine-phosphorylated protein, SIRPα1 [55]. Neuronal SIRPα1 is phosphorylated in response to BDNF [56], and the stimulation of Shp2 by SIRPα1 downstream from TrkB might dephosphorylate FGFR1 and/or its effectors to inhibit differentiation. The inactivation of TrkB signaling due to the overexpression of dominant-negative TrkB may thus suppress Shp2 and release FGFR1 from being inhibited by TrkB-activated Shp2; this, in turn, could lead to enhanced activation of endogenous FGFR1 and the increased axonal filopodial formation that is also observed with bFGF treatment. In contrast, in WT-FGFR1-expressing neurons treated with BDNF, the activation of TrkB may stimulate Shp2, which might then inhibit FGFR1 signaling by dephosphorylating FGFR1 or its effectors to suppress filopodial formation in axons. Intriguingly, we recently found that expression of constitutively active Shp2 [57] but not inactive Shp2 [58] in Xenopus spinal neurons reduced filopodial assembly (Figure S4), which raises the possibility that Shp2 transmits BDNF/TrkB signals. Future studies on the activation of effectors like SIRPα1, FRS2 and Shp2 in spinal neurons may help reveal how bFGF/FGFR1 and BDNF/TrkB signaling pathways blend to trigger and tweak neuronal growth and presynaptic differentiation.

The findings of this study may relate broadly to long-range neuronal pathfinding and target-recognition. An axon crossing a BDNF-rich site may continue to advance, being disinclined to stop due to TrkB activation. But if the axon enters a region where the bFGF level is high, the turning-on of FGFR1 may trigger filopodial extension towards the bFGF source and check axonal advance. Our RT-PCR experiments suggest that bFGF is abundant in muscle but not neural tissue, whereas BDNF and TrkB are both expressed predominantly in neural tissue. Thus, a motor axon's growth before reaching muscle may be spurred on by TrkB auto-activation and/or autocrine BDNF signaling, possibly coupled with low or no background FGFR activity. After a close encounter with a muscle target (and bFGF), intensified FGFR1 signaling in neurons may decelerate axonal growth and initiate changes associated with differentiation. Interestingly, FGFR-signaling is needed for Xenopus RGC axons to recognize their target in the midbrain, the optic tectum, and RGC growth cones avoid FGF-misexpressing cells in vivo [59]. In contrast, BDNF is expressed in the tectum and promotes the branching of RGCs [60], [61], and a BDNF gradient applied in vitro attracts axonal growth towards it [62]. Thus, in the CNS and in the PNS, a reciprocal ramping up and down of NT and FGF signaling may help axons to elongate to their targets and then enable the axons to stop there and establish synapses.

## Methods

### Reagents

BDNF, bFGF and TrkB-FC were purchased from R&D Systems (Minneapolis, MN), K252a from Calbiochem (San Diego, CA), rhodamine-conjugated α-bungarotoxin (R-BTX) was from Molecular Probes (Eugene, OR), and synapsin I antibody and MitoTracker Red were from Sigma (St. Louis, MO).

### Xenopus nerve and muscle primary cultures

Primary cultures of embryonic Xenopus nerve and muscle cells were prepared as described [63]. Briefly, stage 20–22 embryos were dissected and neural tubes and myotomes were separated and dissociated in a Ca^2+^, Mg^2+^-free solution. Dissociated cells were plated on glass coverslips coated with entactin-collagen IV-laminin (E–C–L) substrate (Upstate Biotechnology, Waltham, MA). Neurons were either plated alone or seeded on 7 d-old muscle cultures to prepare nerve-muscle cocultures.

### Visualizing synaptic vesicles

For labeling synaptic vesicles, neuron cultures were fixed in 4% paraformaldehyde in PBS, followed by permeabilization in 0.5% TritonX-100 in 0.2 M NH_4_Cl for 10 min. After washing in PBS, the cultures were blocked with 5% BSA in PBS for more than 1h at room temperature or overnight at 4°C. Then cultures were incubated with synapsin I antibody for 2–3 h at room temperature or overnight at 4°C, washed with PBS for 30 min and labeled with FITC-conjugated secondary antibodies for 45 min at room temperature. After extensive washing in PBS, the coverslips were mounted on glass slides with the anti-bleaching agent Citifluor (Ted Pella, Redding, CA, USA).

To visualize synaptic vesicles in living neurons, mRNAs encoding GFP-synaptophysin, a synaptic vesicle protein tagged with GFP, was microinjected into Xenopus embryos at the two-cell stage. Spinal neurons were isolated from embryos with strong GFP expression and cultured as described above. To visualize mitochondria in neurons, cultures were exposed to medium containing 5 nM MitoTracker Red (Molecular Probes) for 5 min, washed with culture medium and then examined under the microscope.

To visualize live neurons, glass coverslips bearing the neurons were mounted onto custom-made chambers containing culture medium and observed at high magnification with 63x oil objectives. The neurons stayed healthy for 2d under these conditions.

### Ectopic expression of proteins in Xenopus spinal neurons and myotomal muscle cells

Wild-type Xenopus FGFR1 cDNA was a gift from Dr. Robert Friesel (Maine Medical Center Research Institute, USA), wild-type rat TrkB cDNA from Prof. Nancy Ip (HKUST), and cDNA constructs of constitutively active (E76A) and dominant-negative (ΔP) Shp2 in pSP64R1 vector from Dr. Benjamin Neel (Beth Israel Deaconess Medical Center). Human TrkB-T1 (truncated TrkB or TR-TrkB) was cloned from human skeletal muscle cDNA library. Mouse bFGF cDNA was cloned from a mouse heart cDNA library and a truncation-mutant of FGFR1 was prepared by deleting the entire cytoplasmic domain [14]. The cDNAs of wild-type and truncated FGFR1, wild-type and truncated TrkB, and bFGF were all subcloned into pCS2(+) vector and epitope-tagged (C-terminal HA). The mRNAs encoding these tagged exogenous proteins were prepared using an SP6 in vitro transcription kit (mMessage mMachine, from Ambion) and mixed with GFP mRNA at a 6–10∶1 ratio before microinjecting them into 2-cell stage Xenopus embryos using a Drummond Nanojet Oocyte Injector (Drummond Scientific Co., Broomall, PA). Expression of exogenous proteins in cultured cells was confirmed by GFP fluorescence and anti-HA antibody staining [14].

### RT-PCR analysis

Total RNAs were extracted from Xenopus myotomes or neural tubes (with the Trizol reagent from Invitrogen) and 5 µg each of these were used for synthesizing cDNAs with the Superscript III first strand synthesis kit (Invitrogen) and both random and oligo dT18 primers. About 5 ng cDNA was used for 25 cycles of PCR amplification with specific primer pairs for Xenopus bFGF, FGFR1, BDNF, TrkB and GAPDH; the PCR products (200–500 bps long) were resolved by agarose gel electrophoresis and examined. The primers used were: bFGF-F: GCAGGGAGCATCACAACTCT; bFGF-R: GCGGACATTGGGAGAAATAA; FGFR1-F: GCTGCTTTGGGCAAGTAGTC; FGFR1-R: GGTGCAGGCACCAAGTAAAT; BNDF–F: CCCATGAAAGAAGCCAGTGT; BDNF-R: CCTTGCTTTCCTCACTTTGC; TrkB-F: ATGGACCAGATGCAGTCCTC; TrkB-R: CATGCCAAAATCTCCGATCT; GAPDH-F: CAGAGGTGCAGGTCAGAACA; GAPDH-R: GCATCAGCATCAAAGATGGA.

### Immunoprecipitation and immunoblotting

HEK293T cells were cultured in DMEM containing 10 % FBS and cDNAs were transfected into these cells using the calcium phosphate method. For immunoprecipitation (IP) experiments, carried out 24 h after transfection, cells were lysed for 30 min in ice cold IP buffer (Hepes 25 mM pH 7.4, NaCl 150 mM, EDTA 1mM, Triton X-100 1%) containing the protease inhibitor PMSF (1 mM) and the tyrosine phosphatase inhibitor Na-pervanadate (PV; 1 mM). Lysates were clarified by centrifugation, and 30 µl cell extract was mixed with 2X SDS-electrophoresis sample buffer for use as “input”. Various antibodies (1–2 μg) were added to 0.4–0.6 ml extracts along with 15 µl protein A/G agarose slurry; mixtures were then incubated for 3 h at 4°C with end-over-end rotation. The agarose beads were spun down and washed three times with the IP buffer before mixing in 30–50 µl sample buffer to elute the immunoprecipitated proteins.

Denatured proteins were separated by SDS-polyacrylamide gel electrophoresis (8 % gels) and transferred to PVDF membranes for immunoblotting. Membranes were blocked for 1 h with 5 % milk in phosphate-buffered saline containing Tween-20 (PBST) to suppress non-specific antibody binding and then immersed for 1 h in probing solutions, which were primary antibodies diluted in 5% milk-PBST. After washing thrice with PBST (10 min each time), membranes were probed with horseradish peroxidase (HRP)-conjugated secondary antibodies, washed again, and then incubated in enhanced chemiluminescence substrate (West Pico, Pierce) for identifying antibody-stained protein bands.

### Measurement of neuronal growth speed and filopodial assembly and distribution

Axonal growth speeds in 16 h-old cultures were measured by identifying single spinal neurons and photographing them; 1 h later the same neurons were photographed again. Lengths of axons were obtained using MetaMorph software and the differences in lengths between the two time points were calculated and divided by the time interval. To quantify filopodial assembly, segments of axons from neuronal cultures in control or growth factor-containing medium were selected randomly and all filopodia within these segments were counted to calculate filopodial density (#filopodia/10 µm axon). To quantify filopodial asymmetry, axon segments growing within 10–30 µm from the edge of muscle cells were chosen. The asymmetry index (AI) was defined according to the following formula [14]:
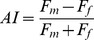
where *F_m_* and *F_f_* are the number of axonal filopodia projecting toward or away from the muscle cell, respectively. AI has the value ranging from −1 to +1. If all filopodia are generated toward muscle, AI is equal to +1. If all filopodia are pointing away from muscle, AI becomes −1. High AI values reflect an increased propensity of neurons to extend filopodia towards muscle [14].

### Quantification of NMJ formation

Live nerve-muscle cocultures were labeled with R-BTX (3 nM; 30 min) to visualize AChR clusters by fluorescence microscopy. NMJ formation was quantified by first identifying nerve-muscle contacts in phase-contrast and then examining these for nerve-induced AChR clusters to calculate percentages of innervation sites with AChR aggregates.

### Microscopy and statistics

To observe live cells at high magnification (63x oil objective lens), glass coverslips with cultured cells were mounted on custom-made sealed chambers; all examinations were completed within 1h after mounting. Images were captured using a ZEISS Axiovert 200 M microscope equipped with a ZEISS AxioCamMR camera. Axiovision Pel 4.5 software was used to control the camera and process images. Data are shown as mean ± SEM and the statistical significance of differences between data sets was assessed by non-paired Student's t-tests (Graphpad Prism statistical software).

## Supporting Information

Figure S1 **Association of presynaptic markers with varicosities along the axon in pure neuron cultures.** (**A–D**) Visualization of synaptic vesicle (SV) clustering in varicosities. Synaptic vesicles were detected by immunolabeling fixed neuronal cultures with synapsin I antibody. Control axons had few varicosities (A and B). After K252a treatment, the distal end of the axon developed into a neuritic web interspersed with varicosities that stained positive for SV antigen synapsin I (arrows in C and D). (E–H) SV clustering was also observed within varicosities in live cultures as an accumulation of GFP-synaptophysin that was ectopically expressed in neurons. Again, control axons showed few varicosities (E and F) but those induced by K252a treatment showed accumulation of this SV marker (arrows in G and H). (I–L) Localization of mitochondria was visualized in live cultures with MitoTrack in control and K252a-treated samples. As in presynaptic nerve terminals, mitochondrial clustering was also seen in varicosities and at the growth cone (arrows).
(TIF)

Figure S2 **Auto-activation of WT-FGFR1 and WT-TrkB after overexpression in HEK293T cells.** (A) HEK293T cells were transfected with cDNAs encoding WT-FGFR1 and TR-FGFR1 proteins with C-terminal HA-tags. From total lysates of transfected cells, WT-FGFR1 and TR-FGFR1 were immunoprecipitated with the anti-HA antibody and immunoblotted with anti-HA (upper blot) and anti-phosphotyrosine mAb 4G10 antibodies (lower blot). Staining by anti-phosphotyrosine showed that WT-FGFR1 was auto-activated (as evidenced by the phosphotyrosine signal) but that TR-FGFR1 was not. (B) HEK293T cells were transfected with cDNAs encoding WT-TrkB and TR-TrkB (again HA-tagged). WT-TrkB and TR-TrkB were immunoprecipitated from total lysates with anti-HA antibody and immunoblotted with anti-HA (upper blot) and mAb 4G10 (lower blot). Staining by anti-phosphotyrosine showed that WT-TrkB could become tyrosine phosphorylated (and thus auto-activated) but that TR-TrkB could not. The positions of MW markers are indicated on the right-hand-side of the blots. IP: immunoprecipitation; WB: western blot.
(TIF)

Figure S3 **The influence of BDNF bath application on the asymmetric extension of axonal filopodia towards muscle cells.** Nerve-muscle cocultures were treated (A) without or (B) with BDNF. In control cocultures (A; Ctl), neurons extruded filopodia preferentially from their muscle-facing side, but when BDNF was added (B) the neurons tended to extend less filopodia and they also showed a reduced preference to send out filopodia from their muscle-facing side. (C) Calculation of AI values showing that asymmetric extension of filopodia was reduced in BDNF-treated cocultures. Mean and SEM shown; t test: *p<0.05, compared to Ctl cocultures.
(TIF)

Figure S4 **Effect of Shp2 activation on axonal filopodial formation.** Pure nerve cultures were prepared using GFP-expressing neurons (Ctl) or neurons overexpressing constitutively active Shp2 (E76A) or dominant-negative Shp2 (together with GFP). E76A-Shp2-expressing neurons (C and D) grew less filopodia than neurons expressing Δp-Shp2 (E and F) or GFP (A and B). (G) Axonal filopodial densities were calculated and normalized relative to that of GFP-neurons. E76A-Shp2-neurons had >50% fewer filopodia than GFP-neurons, whereas Δp-Shp2-expressing neurons had a similar filopodial density as control neurons. Mean and SEM shown; t test: **p<0.01, compared to Ctl cocultures.
(TIF)
