# Supplementary figures and images for: Reciprocal Regulation of Axonal Filopodia and Outgrowth during Neuromuscular Junction Development
Source: PLoS One. 2012 Sep 5;7(9):e44759. doi: 10.1371/journal.pone.0044759 (PMC3434160; doi:10.1371/journal.pone.0044759)

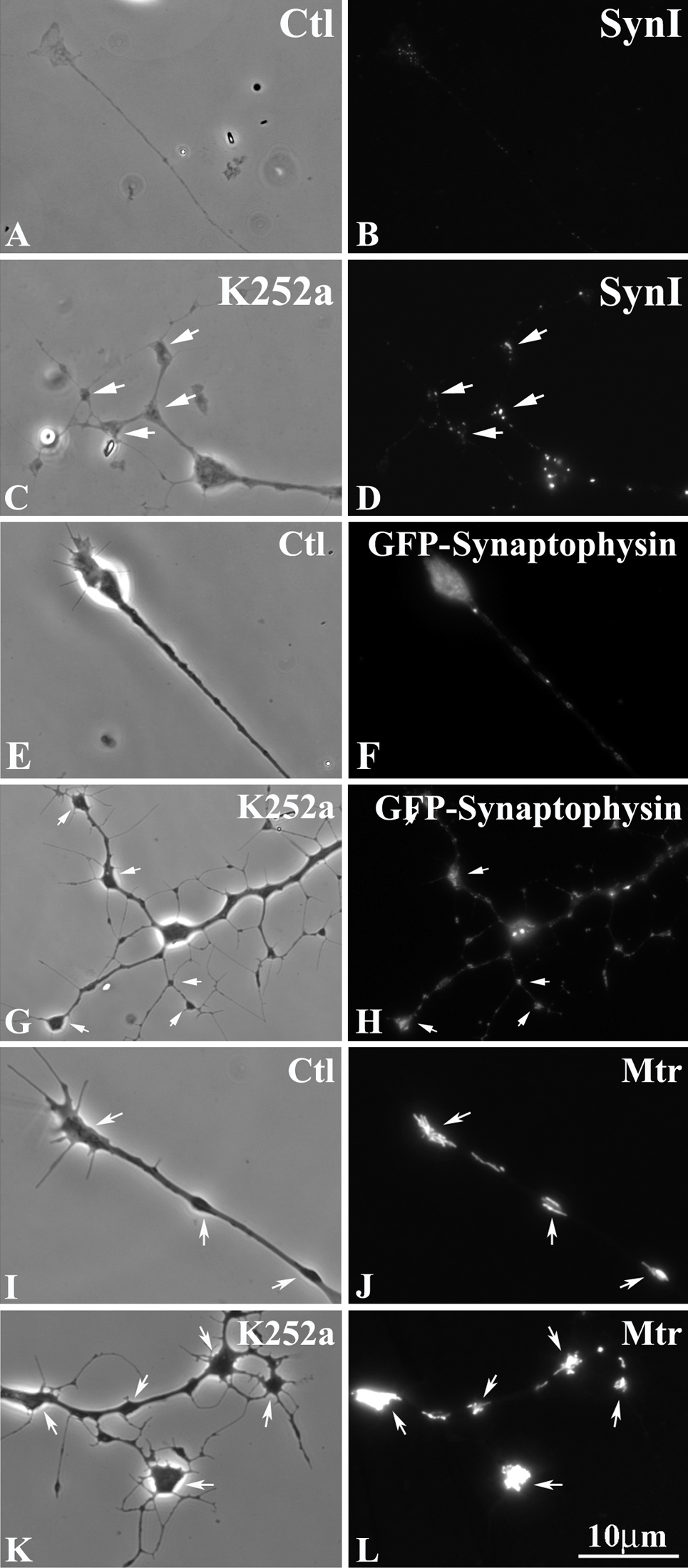

Supplement: Figure S1 — Association of presynaptic markers with varicosities along the axon in pure neuron cultures. (A–D) Visualization of synaptic vesicle (SV) clustering in varicosities. Synaptic vesicles were detected by immunolabeling fixed neuronal cultures with synapsin I antibody. Control axons had few varicosities (A and B). After K252a treatment, the distal end of the axon developed into a neuritic web interspersed with varicosities that stained positive for SV antigen synapsin I (arrows in C and D). (E–H) SV clustering was also observed within varicosities in live cultures as an accumulation of GFP-synaptophysin that was ectopically expressed in neurons. Again, control axons showed few varicosities (E and F) but those induced by K252a treatment showed accumulation of this SV marker (arrows in G and H). (I–L) Localization of mitochondria was visualized in live cultures with MitoTrack in control and K252a-treated samples. As in presynaptic nerve terminals, mitochondrial clustering was also seen in varicosities and at the growth cone (arrows). (TIF) [file pone.0044759.s001.tif]

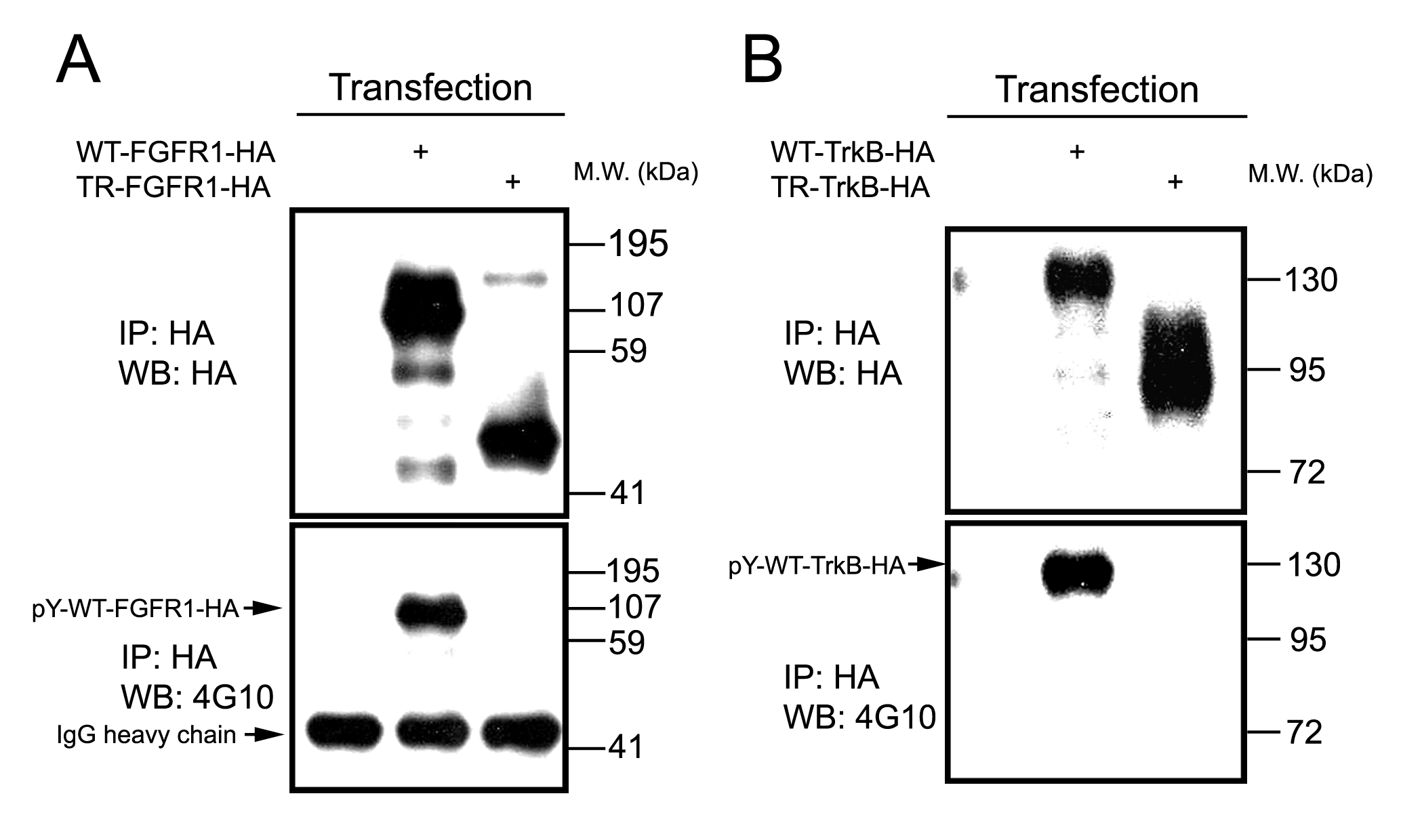

Supplement: Figure S2 — Auto-activation of WT-FGFR1 and WT-TrkB after overexpression in HEK293T cells. (A) HEK293T cells were transfected with cDNAs encoding WT-FGFR1 and TR-FGFR1 proteins with C-terminal HA-tags. From total lysates of transfected cells, WT-FGFR1 and TR-FGFR1 were immunoprecipitated with the anti-HA antibody and immunoblotted with anti-HA (upper blot) and anti-phosphotyrosine mAb 4G10 antibodies (lower blot). Staining by anti-phosphotyrosine showed that WT-FGFR1 was auto-activated (as evidenced by the phosphotyrosine signal) but that TR-FGFR1 was not. (B) HEK293T cells were transfected with cDNAs encoding WT-TrkB and TR-TrkB (again HA-tagged). WT-TrkB and TR-TrkB were immunoprecipitated from total lysates with anti-HA antibody and immunoblotted with anti-HA (upper blot) and mAb 4G10 (lower blot). Staining by anti-phosphotyrosine showed that WT-TrkB could become tyrosine phosphorylated (and thus auto-activated) but that TR-TrkB could not. The positions of MW markers are indicated on the right-hand-side of the blots. IP: immunoprecipitation; WB: western blot. (TIF) [file pone.0044759.s002.tif]

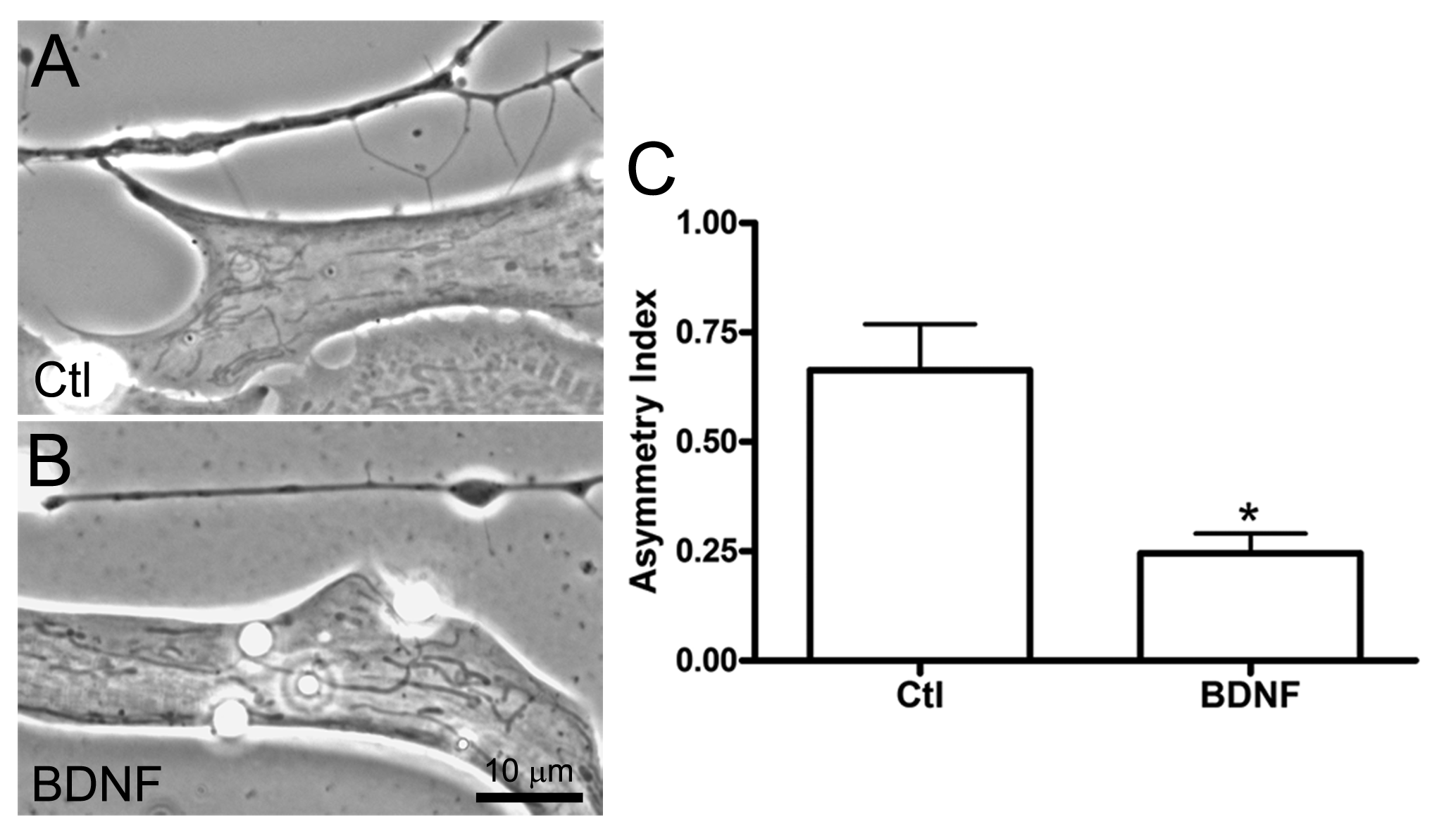

Supplement: Figure S3 — The influence of BDNF bath application on the asymmetric extension of axonal filopodia towards muscle cells. Nerve-muscle cocultures were treated (A) without or (B) with BDNF. In control cocultures (A; Ctl), neurons extruded filopodia preferentially from their muscle-facing side, but when BDNF was added (B) the neurons tended to extend less filopodia and they also showed a reduced preference to send out filopodia from their muscle-facing side. (C) Calculation of AI values showing that asymmetric extension of filopodia was reduced in BDNF-treated cocultures. Mean and SEM shown; t test: *p<0.05, compared to Ctl cocultures. (TIF) [file pone.0044759.s003.tif]

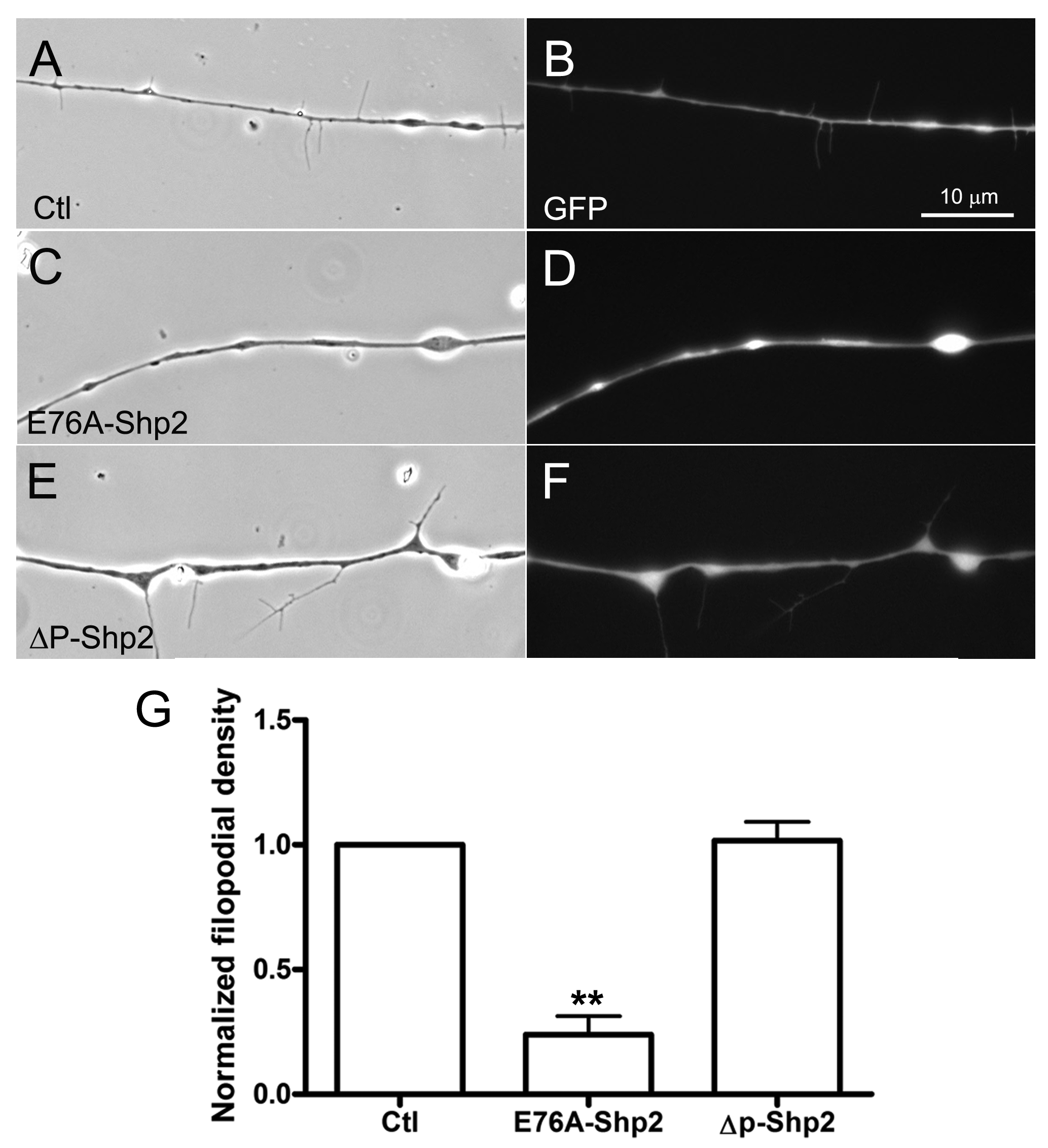

Supplement: Figure S4 — Effect of Shp2 activation on axonal filopodial formation. Pure nerve cultures were prepared using GFP-expressing neurons (Ctl) or neurons overexpressing constitutively active Shp2 (E76A) or dominant-negative Shp2 (together with GFP). E76A-Shp2-expressing neurons (C and D) grew less filopodia than neurons expressing Δp-Shp2 (E and F) or GFP (A and B). (G) Axonal filopodial densities were calculated and normalized relative to that of GFP-neurons. E76A-Shp2-neurons had >50% fewer filopodia than GFP-neurons, whereas Δp-Shp2-expressing neurons had a similar filopodial density as control neurons. Mean and SEM shown; t test: **p<0.01, compared to Ctl cocultures. (TIF) [file pone.0044759.s004.tif]
